# High-precision automated bone age: a clinically useful tool in monitoring of treatment effects in children and adolescents

**DOI:** 10.1038/s41598-026-49670-1

**Published:** 2026-04-27

**Authors:** Hans Henrik Thodberg, Lise Aksglaede, Anders Juul, Shanlee M. Davis, Judith Ross

**Affiliations:** 1 Visiana, Fremtidsvej 1, 2970 Hørsholm, Denmark; 2 Department of Growth and Reproduction, Copenhagen University Hospital-Rigshospitalet, Copenhagen, Denmark; 3 International Centre for Research and Research Training in Endocrine Disruption of Male Reproduction and Child Health (EDMaRC), Rigshospitalet and University of Copenhagen, Copenhagen, Denmark; 4 Department of Clinical Medicine, University of Copenhagen, Copenhagen, Denmark; 5 Department of Pediatrics, Section of Endocrinology, University of Colorado School of Medicine, Aurora, CO USA; 6 Department of Pediatrics, Nemours Children’s Hospital-DE and Thomas Jefferson University, Philadelphia, PA 19107 USA

**Keywords:** Bone age, Greulich-Pyle, Bone density, Klinefelter syndrome, Diseases, Health care, Medical research

## Abstract

The clinical value of serial bone age (BA) determinations in children during growth is limited by the manual rater variability (precision 0.63 years). The objective of this work was to determine the precision of automated bone age and bone health index (BHI) measurements by BoneXpert and to establish the time interval at which the automated method can detect a significant treatment effect. The data were from a case-control trial (oxandrolone/placebo) following 90 boys with Klinefelter syndrome (KS) with five visits over 2 years, recording X-rays of both hands. The precision of BA was 0.08 years [0.07; 0.09] 95% CI, leading to a minimal detectable difference of 0.23 years. The effect of androgen treatment on BA was 0.24, 0.77, 1.24 and 1.43 years after 6, 12, 18 and 24 months, respectively. Thus, the effect on BA was detectable by 6 months. In conclusion, automated BA determination is markedly more precise, compared to manual X-ray readings and can detect 0.23 years changes. Automated BA is clinically useful in follow-up in children and adolescents during growth-modulating therapies.

## Introduction

Assessment of skeletal maturity or bone age (BA) is routinely used to evaluate children and adolescents with disorders of growth and development. BA can be used to estimate the growth reserve and predict final adult height. In addition, repeated assessment of BA is used to monitor the long-term growth-modulating effects of interventions like growth hormone, sex steroids or gonadotropin-releasing hormone (GnRH) analog, but the considerable inter- and intra-rater variability of manual BA assessments has limited the clinical value of frequent evaluations. The high variability of manual BA assessment has led to substantial controversy regarding the optimal use of BA determinations in clinical practice^1,2^.

To overcome the rater variability, the automated BA method BoneXpert was developed and launched in Europe in 2009 as a medical device^3,4^. In 2019, BoneXpert version 3^5^ extended the tool to cover the entire BA range of 0–19 years based on the Greulich–Pyle (GP) method^6^. BoneXpert’s primary BA analysis is computed from the 21 tubular bones: radius, ulna, metacarpals and phalanges. Additionally, BoneXpert provides a separate assessment of BA for the carpals (carpal BA) and Tanner-Whitehouse 3 BA (TW3 BA) from 13 tubular bones^7^. BoneXpert does not calculate carpal BA beyond about 9 years for girls and 11 years for boys due to the increasing overlap among the carpal bones, which hinders accurate shape analysis.

In addition to BA, BoneXpert also measures the cortical thickness T of the metacarpals and combines this with the metacarpal length and width to form the Bone Health Index (BHI)^8^, a metric related to bone density. Based on BHI reference curves versus BA, BoneXpert also estimates the BHI standard deviation score (BHI SDS).

It is important to distinguish between the *accuracy* of a bone age rating (the ability to determine the true value) and the *precision* (the ability to obtain the same value in repeated measurements). Accuracy is the most relevant quality metric for a BA method used during a child’s initial evaluation to assess whether the child is delayed or advanced and to predict adult height based on the BA. The BA accuracy is quantified as the root mean square (RMS) of the deviations between the predicted bone age and the true value. The true value is defined as the average of multiple (in principle infinitely many) manual ratings of the image. The accuracy of BA by BoneXpert was determined to be an RMS error of 0.33 years^5^. This was derived from a study of 200 images rated by six raters, where the observed RMS error was 0.45 years relative to the average of these six ratings. Statistical analysis projected that with an infinite number of ratings, the error would decrease to 0.33 years.

The BA precision is quantified as the SD of BA assessments from repeated X-ray images taken on the same day. Precision becomes the relevant quality metric when BA is used to monitor change over time, as the focus is on assessing BA *increments*, i.e., the changes in BA from one visit to the next. Therefore, monitoring the effects of an intervention on BA requires high precision. The precision of *manual* BA ratings has been studied extensively^9,10^. These studies typically rely on multiple human evaluations of the same X-ray, implicitly assuming that the variability introduced by acquiring a new image is negligible. The precision error of human ratings varies depending on the experience of the raters and the time devoted to each rating. In a study using 16 images rated by 12 observers, the precision of manual ratings was estimated at 0.63 years^11^. Thus, manual BA reading is very imprecise, limiting its use. Since the true rating is defined as the average of multiple human ratings, human accuracy is by definition equal to human precision. The precision of BoneXpert BA has been studied on images recorded on film in the 1960s^12^, but not on modern images recorded digitally. When determining the precision of an automated method, it is essential to include the recording of a new X-ray, as repeated application of an automated method like BoneXpert to the same digital image yields an identical value, because there is no randomness in the algorithm.

The aim of this study was to evaluate the precision and clinical utility of automated, repeated BA assessment in monitoring the effect of treatments. Specifically, we sought to determine the minimal time interval between BA exams required for the automated method to detect a significant treatment effect. The data for this study were drawn from a previously conducted intervention study^13^ involving 90 boys with Klinefelter syndrome (KS) in which images of both the left and right hands were obtained semiannually over 2 years, enabling determination of precision for the automated BA assessment. The present study is not focused on the use of bone age in children with KS in particular, but rather in its use in pediatric endocrinology in general.

## Materials and methods

### Study population and design

A total of 93 boys with KS aged 4–12.9 years participated in a double-blind placebo-controlled randomized trial performed at Thomas Jefferson University in Philadelphia, Pennsylvania from 2007 to 2011. In three boys, no X-rays were available, leaving a total of 90 boys for the purpose of this study. Details of the study have previously been published^14,15^. In brief, the participants were randomized 1:1 to treatment for 2 years with oral oxandrolone (a nonaromatizable anabolic steroid) or placebo. Standing height and X-rays of the left and the right hands were performed biannually. Participants missed forty-nine visits, leading to a total of 401 visits with available X-ray images. BA and BHI values for this sample, as determined by BoneXpert version 2, have previously been published^13^. For the current study the images were reanalyzed using BoneXpert version 3 (Visiana, Hørsholm, Denmark).

**Fig. 1:**
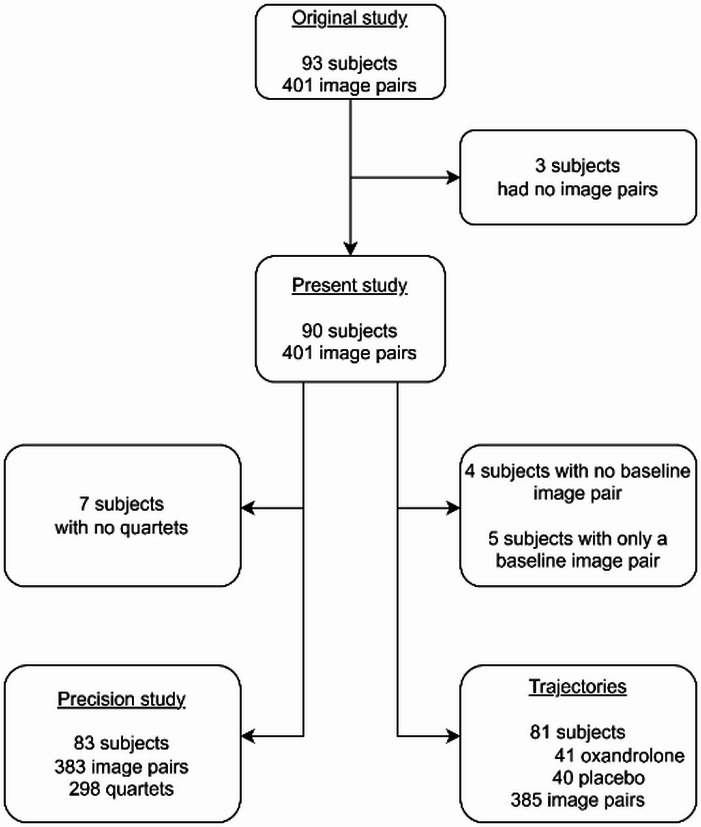
Flowchart of data selection.

The data were used for two analyses based on slightly different image selections, as outlined in Fig. 1:

- The determination of the precision was based on quartets of images, i.e., two left-right pairs of images taken at subsequent visits (explained in detail below).
- The study of trajectories was based on subjects with images taken at baseline and at least one later visit.

### Ethical approval

The study was approved by the Human Subjects Committee of Thomas Jefferson University. Written informed consent was obtained from parents or legal guardians, and assent was provided by the participating children.

The study was performed in accordance with all relevant guidelines and regulations.

### The automated method: BoneXpert version 3

This software medical device was trained to agree with manual GP BA rating based on 33,000 images from seven sites in Europe and the USA. The software uses machine learning (not deep learning) to implement a regression, i.e., it converges to predict what would be the average manual ratings of the images, i.e., the true rating, and the method arrives at predicting the true rating more accurately than a single manual rater, as previously reported in detail^5^.

Predicted adult height was also determined based on age, BA, sex and height^16^.

### Estimation of precision by the quartet method

To evaluate the precision of the automated method, an ideal study would involve multiple X-rays of the same child on the same day. Since such study data were not available, we developed a method to derive the precision from two visits where both the left and the right hands were imaged. The bone age *increment* is expected to be the same in the left and the right hand, and if the observed increments are *not* the same, it is interpreted as an effect of the precision error of the bone age method.

The method is termed the quartet method, and it enables an assessment of the precision from the serial X-rays of the existing study. The method uses quartets of measurements derived from images from the same participant taken of the right and left hands at two consecutive visits. The study design had five visits, allowing for four quartets, namely from visits 1 + 2, 2 + 3, 3 + 4 and 4 + 5, for each participant.

Consider the BA increment in the same hand between two visits, 1 and 2:

$$\Delta {\mathrm{BA}}={\mathrm{B}}{{\mathrm{A}}_{\mathrm{2}}}-{\mathrm{B}}{{\mathrm{A}}_{\mathrm{1}}}$$

The quartet error *q* is defined as the difference between the increments in the left and right hands:

$$q=\Delta {\mathrm{B}}{{\mathrm{A}}_{{\mathrm{left}}}}-\Delta {\mathrm{B}}{{\mathrm{A}}_{{\mathrm{right}}}}$$

Bone maturation is a systemic process controlled by circulating hormones. Because hormone concentrations are identical on both sides of the body, the tempo of bone maturation is expected to be the same in the left and right hands. The quartet method assumes that a difference in the observed BA increments in the left and right sides is due to precision error. The RMS of *q* is denoted *s*_*q*_, and it has contributions from the measurement errors of all the four measurements in the quartet. Assuming that these errors are independent, the variance of *q* is four times the variance of the individual measurement errors, so the precision error *p*, defined as the standard deviation on single BA measurements, can be estimated as.

$$p={s_q}/2$$

If the measurement method has a bias resulting in a consistently higher BA in the left hand than the right hand, the quartet method is insensitive to this.

To observe a significant change in a parameter such as BA, the change must be at least 2√2 times the precision error *p*. This is because the SD of an increment is √2 *p*, so a 2-SD-effect corresponds to approximately 95% confidence that this is not a random finding due to measurement error (using 2 rather than 1.96 for simplicity). Thus, the smallest detectable change is calculated as 2√2 *p.*

The quartet method was also used to assess the precision of BHI. It is expected that the dominant hand has slightly higher BHI than the non-dominant hand, but again, the quartet method is insensitive to such a systematic difference.

### Statistics

The quartet method was used to determine the precision of the GP BA (primary outcome), carpal bone age, TW3 BA, each individual bone bone-age, and BHI determined by BoneXpert. Confidence intervals and two-tailed p-values were computed using non-parametric bootstrapping. The bootstrapping was done by resampling subjects, to take into account that the quartets formed from the same subject were not statistically independent.

## Results

**Fig. 2:**
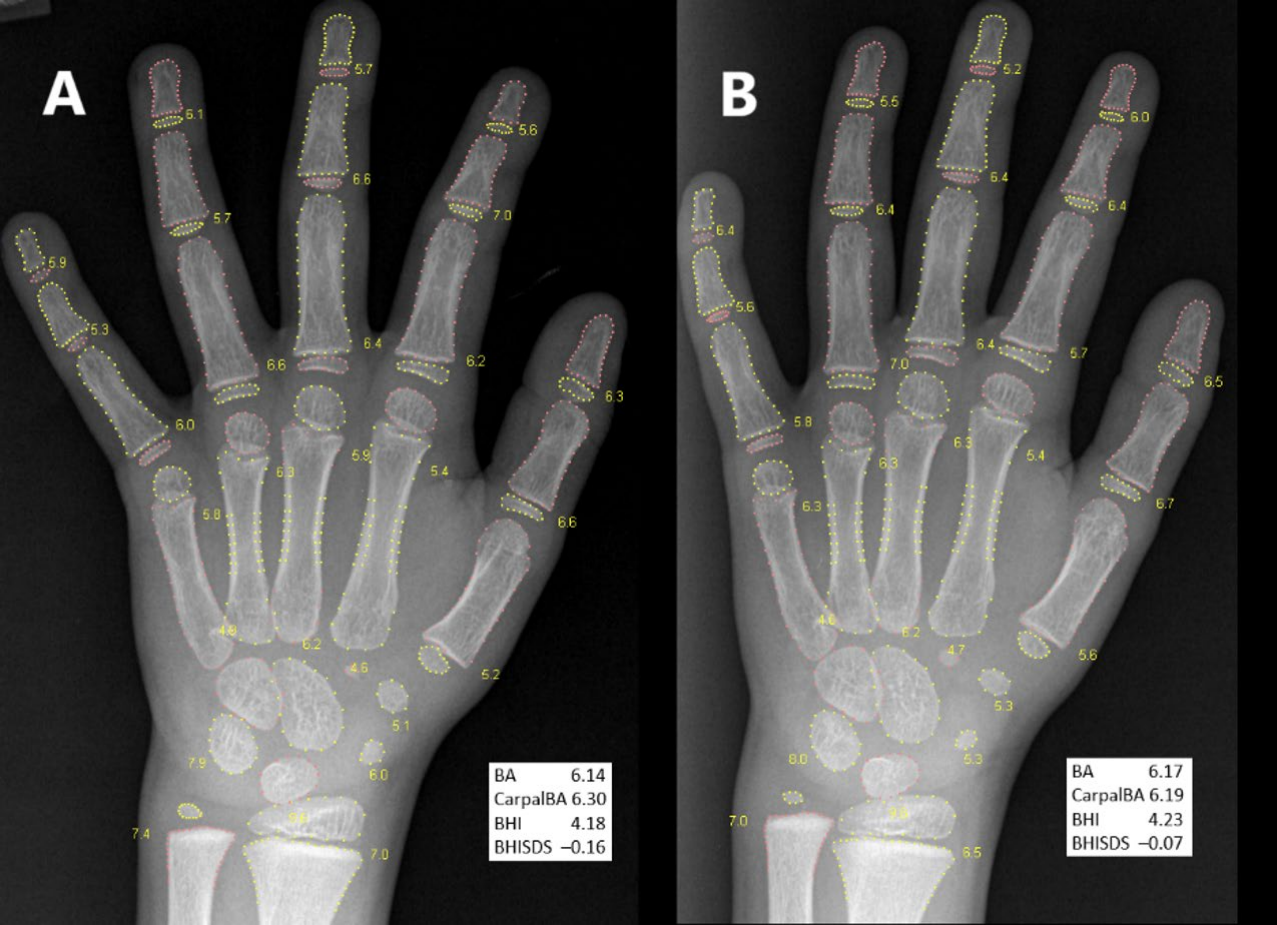
Posterior-anterior X-rays of the left (**A**) and right (**B**) hands of one participant obtained at the same visit. The right hand is shown mirrored. BoneXpert localizes the bone borders as indicated by the dotted lines, which are drawn in alternating yellow and red colors to aid the eye. The BoneXpert Greulich–Pyle bone ages of the individual bones are indicated next to the 21 tubular and 7 carpal bones. In the white boxes, the software has calculated the overall average bone age from the 21 tubular bones (BA) (standard Greulich–Pyle method) as well as an average from the carpal bones (carpal BA). In the shafts of the three middle metacarpals, BoneXpert’s localization of the cortex is indicated, which is used to compute the bone health index metric (BHI), and its Standard Deviations score (BHI SDS).

X-ray images of the left and right hand from the same participant at one visit are shown in Fig. 2.

**Fig. 3:**
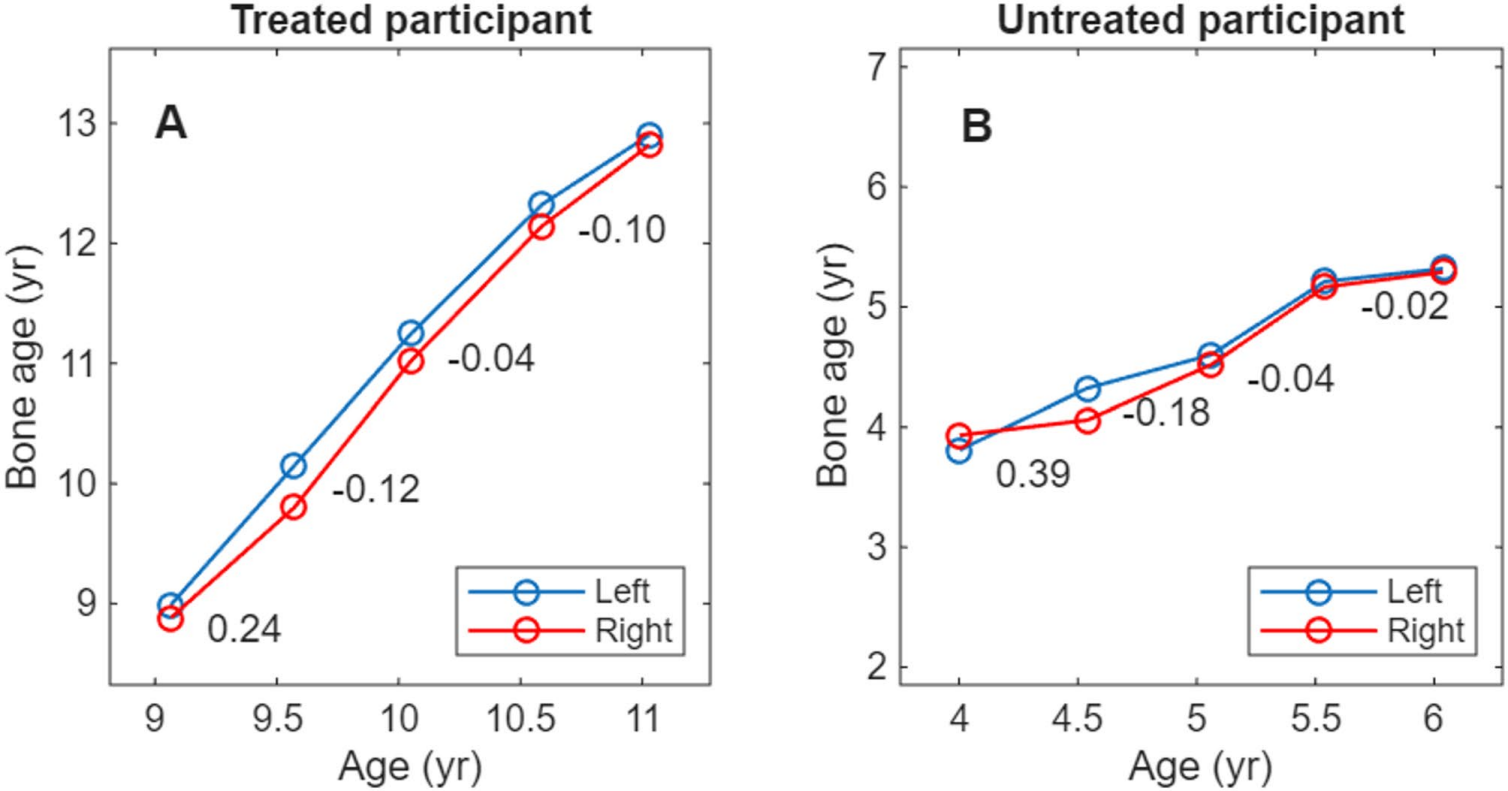
Longitudinal series of bone age assessments of the left and right hands for a participant on treatment **(A**) and an untreated participant (**B**). The quartet errors are indicated for the four quartets that were formed for each of these participants. From the first to the second visit in B, the bone age increments were somewhat different, so here the quartet error was relatively large: 0.39 years. The precision derived solely from participant A or B (as half the rms of the quartet errors) were 0.07 years and 0.11 years respectively, representing typical cases.

Longitudinal data on BA from two participants are presented in Fig. 3.

### Precision of automated BA assessments by different BA methods

BoneXpert was able to determine GP BA, TW3 BA and BHI on both hands for all 401 visits, which allowed a total of 298 quartets to be formed. The difference between the BA in the left and right hands at the same visit was 0.018 (0.150; [− 0.006; 0.040]) years (mean (SD; [95% CI])), thus there was no significant difference between BA in the left and right sides.

Carpal BA was determined up to a carpal BA of approximately 10.5 years, and there were 191 quartets available for determination of the precision of carpal BA.

The mean BA of the 298 quartets (defined as the average over its four members) was 8.1 years (SD 3.0; range 2.9–15.0).

The precision of GP BA, carpal BA, and TW3 are summarized in Table 1. For GP BA the precision was 0.08 years, so the smallest detectable change of BA was 2√2 *p* = 0.23 years.

**Table 1:** Precisions of bone age: The precision determined by the quartet method for the three types of bone age studied.

Bone age type	Quartets	Precision with 95% confidence interval	Smallest detectable change
GP BA	298	0.08 years [0.07; 0.09]	0.23 years
Carpal BA	191	0.15 years [0.13; 0.17]	0.42 years
TW3 BA	298	0.17 years [0.15; 0.19]	0.48 years

GP BA is the Greulich–Pyle bone age derived from the 21 tubular bones, carpal BA is for the up to seven carpal bones, and TW3 BA is the Tanner Whitehouse 3 BA.

To analyse the precision further, Table 2 shows the precision when the quartets were divided into tertiles of BA. The precision was better in the upper tertile compared to the middle. The table also reports the precision of treated and untreated participants separately, showing no significant difference.

**Table 2:** Precision of bone age in subgroups: The first three subgroups are the tertiles of BA, while the last two divided the data into treated and placebo participants.

Subgroup	Quartets	BA range (years)	Average BA (years)	Precision with 95% confidence interval (years)
Lower BA tertile	99	2.8–6.8	5.3	0.077 [0.068; 0.087]
Middle BA tertile	100	6.8–10.5	8.6	0.090 [0.080, 0.101]
Higher BA tertile	99	10.5–15.8	12.4	0.063 [0.056; 0.072]
Treated	148	2.8–14.2	8.5	0.076 [0.068; 0.084]
Placebo	150	2.9–15.8	9.0	0.079 [0.071; 0.088]

The precision in the higher BA tertile was better than in the middle tertile (but this difference should not be considered statistically significant due the many potential comparisons one could make).

Finally, the images were also analysed as females. The BA range was 3–12 years, and the observed precision was 0.08 years, the same as when analysed as boys. This tests the female “branch” of the algorithm. It is not a genuine assessment of the precision in girls, only an approximation, or a simulation.

### Precision of single-bone bone age

**Fig. 4:**
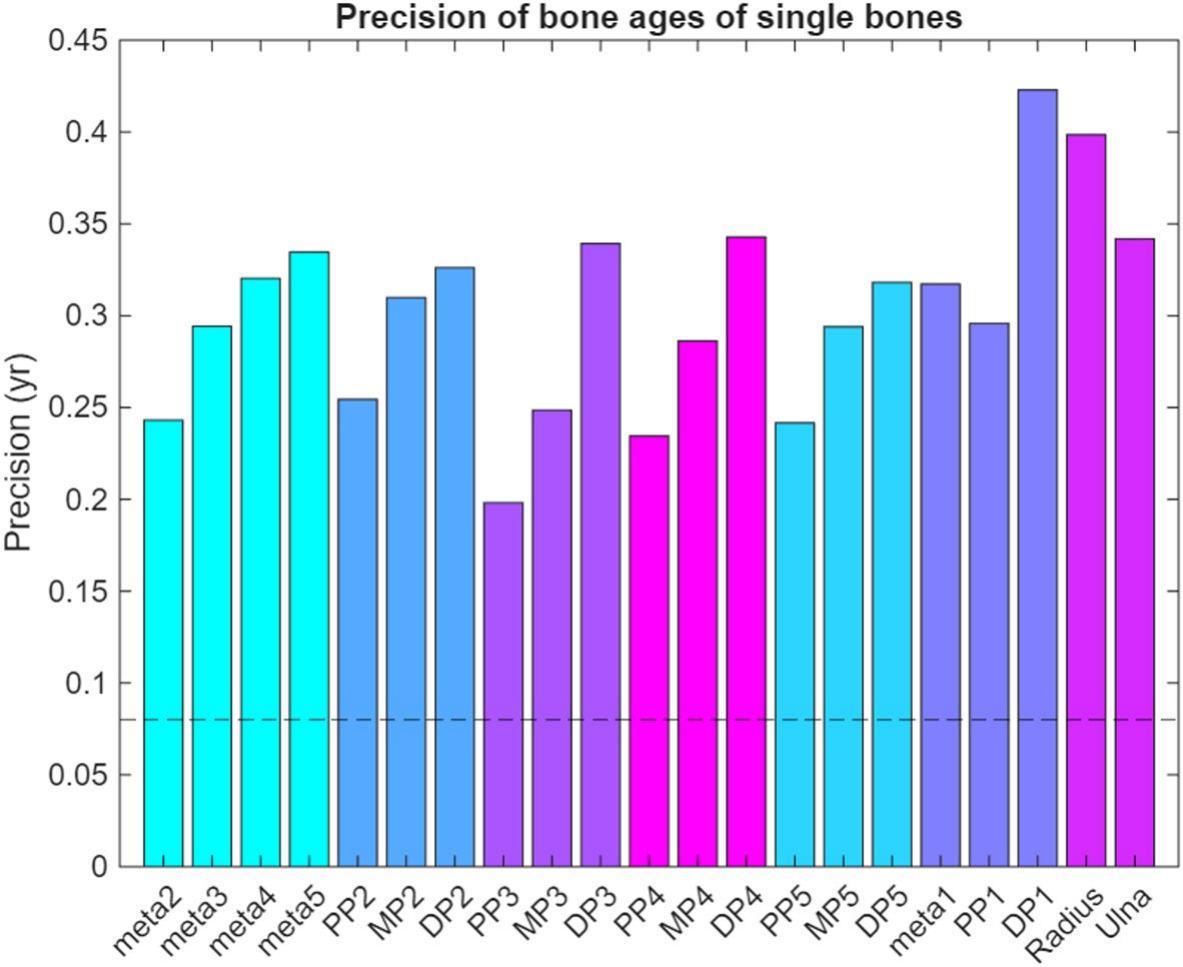
Precision of the bone ages of single bones, where meta refers to metacarpals, PP to proximal phalanges, MP to middle phalanges and DP to distal phalanges. Thus, PP2 is the proximal phalanx of the index finger. The dashed line indicates the precision of the full BA.

The precision error of the single-bone BAs varied from 0.20 to 0.42 years, with an RMS of 0.31 years. It increased from metacarpals 2–5 as well as from proximal to distal within each phalanx. The most precise was the proximal phalanx of digit 3 (PP3). The distal phalanx of the thumb (DP1) exhibited the poorest precision, followed by radius and then ulna (Fig. 4).

### Precision of bone health index

For BHI, the mean (SD; [95% CI]) difference between the right and left side was 0.64% (3.9% [− 0.12%; 1.34%]), i.e., the right hand had 0.64% higher BHI than the left (not significant, *p* = 0.06).

The precision of BHI was found to be 1.6% [1.4; 1.8]. When dividing the quartets into bins of average cortical thickness, the precision of BHI—expressed in percent—was consistent with being inversely proportional to the cortical thickness. Therefore, it is more appropriate to report the BHI precision as a function of cortical thickness as shown in Table 3. The precision of the cortical thickness itself was found to be 23 microns, independent of the cortical thickness.

**Table 3:** Precision of Bone Health Index (BHI).

T (mm)	Approximate age (years)	BHI precision (%)	Smallest detectable change (%)
0.6	1	3.0	8.5
1.0	5	1.8	5.1
1.3	10	1.39	3.9
1.8	13	1.00	2.8
2.0	16	0.90	2.5

The precision of BHI varies with the cortical thickness (T). The age at which healthy subjects on average attain this cortical thickness is indicated.

The BHI reference curves used to derive BHI SDS entail a constant SD of 7.5%, so BHI SDS has a precision of 0.21 units, which multiplied by 2√2 yields 0.60 as the smallest detectable change in BHI SDS.

### Increments of bone age, bone health, and predicted adult height during treatment

**Fig. 5:**
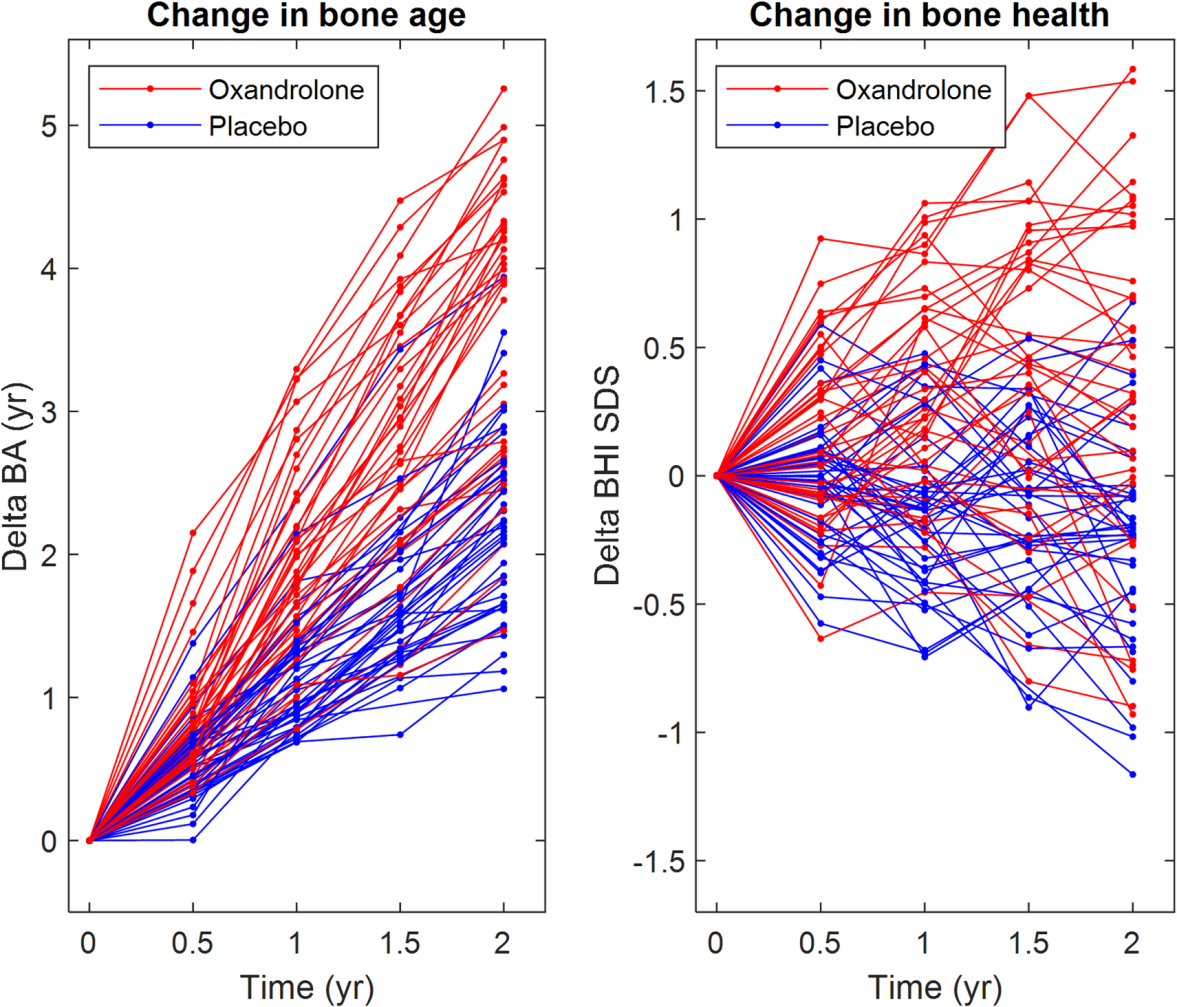
Individual changes in bone age (left panel) and bone health index (right panel) in 81 participants with Klinefelter syndrome followed every 6 months for 2 years. Each broken line represents one participant; red lines indicate treatment with oxandrolone, and blue lines indicate treatment with placebo.

**Fig. 6:**
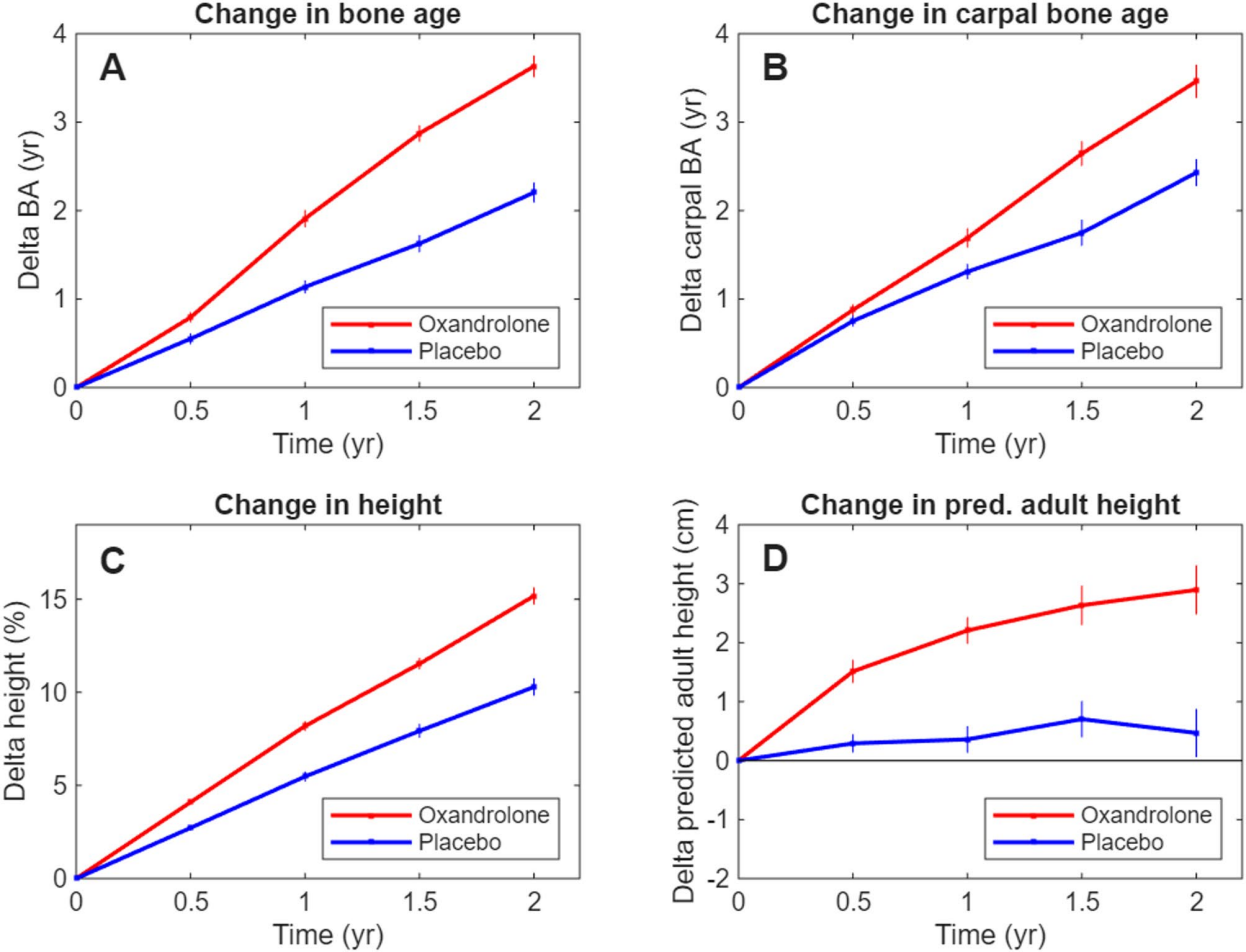
Average trajectories of treated and untreated subjects. The error bars indicate the SEs of the mean, which serve as guidance to appreciate when these average treatment effects are statistically significant (in contrast, Table 4 reports the SDs across subjects).

**Fig. 7:**
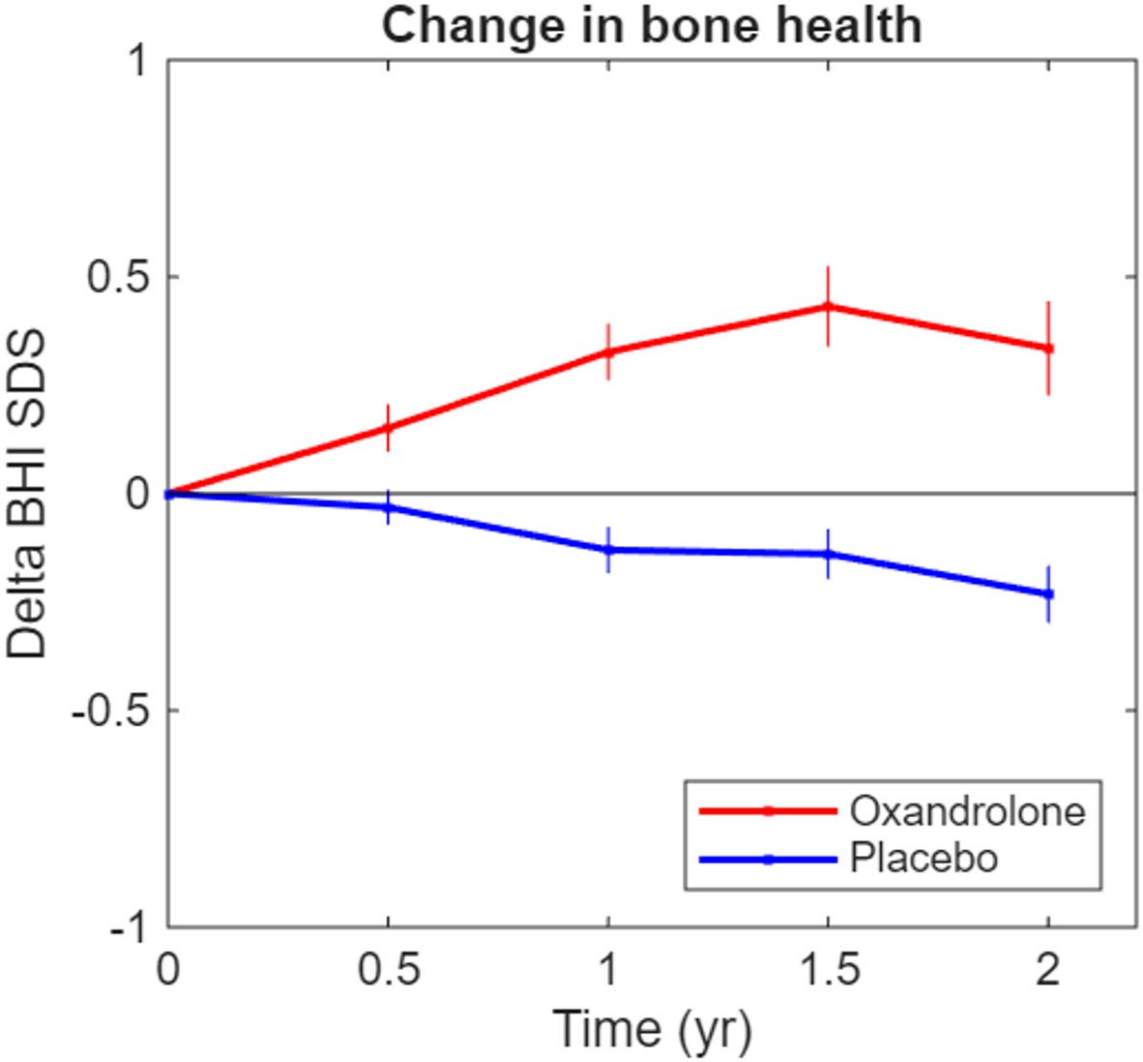
Average trajectories of BHI SDS in treated and untreated subjects. Error bars indicate SEs of the means.

Individual changes in BA and BHI SDS for each participant with or without treatment are shown in Table 4; Fig. 5. The participants treated with oxandrolone had a larger increase in both BA and BHI SDS, but with some variability within each group. The mean changes in BA, carpal BA, height, predicted adult height and BHI SDS from baseline are shown in Figs. 6 and 7. After 2 years of treatment, the BA advancement in the group treated with oxandrolone relative to the placebo group had reached + 1.4 years. The carpal BA response to treatment was smaller, reaching an advancement of 1.0 year after 2 years of treatment (Fig. 6B).

**Table 4:** Change in BA and BHI SDS from baseline in this cohort.

	Placebo			Oxandrolone		
Period	N	Mean (years)	SD (years)	N	Mean (years)	SD (years)
Bone age						
0–6 months	39	0.55	0.38	39	0.79*	0.36
0–12 months	38	1.14	0.46	41	1.91**	0.60
0–18 months	35	1.63	0.62	35	2.87**	0.58
0–24 months	38	2.20	0.70	39	3.63**	0.76
BHI SDS						
0–6 months	39	− 0.03	0.25	39	0.15*	0.34
0–12 months	38	− 0.13	0.33	41	0.33**	0.40
0–18 months	35	− 0.14	0.36	35	0.43**	0.58
0–24 months	38	− 0.23	0.41	39	0.34**	0.67

The mean and SD of the changes from the baseline for the untreated (placebo) and treated (oxandrolone) groups. The first table is for bone age changes, the second for changes in BHI SDS. One or two stars on the oxandrolone values indicate that the means were significantly different from placebo with *p* < 0.05 and *p* < 0.01 respectively.

Height increased by 16% in treated versus 10% in placebo after 2 years (Fig. 6C).

Predicted adult height increased by 2.9 cm in the treated group and 0.4 cm in the placebo group (Fig. 6D).

## Discussion

In this study, we reanalyzed 401 pairs of X-rays of the left and the right hand from 90 boys with KS, who had participated in a double-blind, placebo-controlled randomized trial, using BoneXpert version 3 to determine the precision error of automated BA and BHI assessments. We found a very low precision error of 0.08 years for automated BA determination, much smaller than a human rater precision of 0.63 years (this human rater precision was derived in a study where humans rated the same X-ray. Had they used repeated X-rays, the precision could have been slightly worse). The precision was found to be better in the upper BA tertile compared to the middle tertile. This may indicate that the maturity indicators change more rapidly when bone age surpasses 10.5 years.

Several methods to calculate bone age (e.g. Greulich–Pyle bone age, carpal bone age, TW3 bone age) exist and can all be determined simultaneously using BoneXpert. Interestingly, the precision error of TW3 BA (0.17 years), was considerably larger than for GP BA. An explanation for this is that while GP BA assigns 1/21 weight to each bone, TW3 BA uses only 13 bones and assigns 1/5 weight to radius, 1/5 to ulna, 1/15 to each of the three bones in the thumb (ray 1), and 1/20 to each of the four bones in ray 3 (the middle finger plus metacarpal 3) and ray 5. An imprecision in radius or ulna will therefore have a relatively large impact on the imprecision of TW3 BA. Carpal BA was less precise (0.15 years) than the tubular BA, which is plausible as it is the average over fewer bones, namely up to seven carpals. Also, the shape changes in carpals are less pronounced than for the tubular bones, which could also lead to poorer precision.

The precision of the single-bone BAs varied from 0.20 to 0.42 years, with a RMS of 0.31 year. If the precision errors were independent among the 21 bones, the precision of their mean value would be 0.31 years/sqrt(21) = 0.07 years, close to the observed precision of 0.08 years. This shows that averaging over many bones is the key to good precision.

The precision of bone age as determined with BoneXpert version 2 has previously been reported to be 0.18 years in a study of left-right pairs of films recorded in 1955–1975^17^. That study reported no difference in the average left- and right-hand BA, as was also found in the present study. There are three explanations for the better precision determined in the present study. Firstly, in the old study, precision was determined using the doublet method, which computes the RMS difference between the left- and right-hand BAs and divides by √2. This method gives a larger estimated precision error because it does not compensate for any systematic BA difference between the hands. To quantify this effect, we also analyzed our dataset using the doublet method with version 3, yielding a precision of 0.11 years, i.e., an increase of 0.03 years. Secondly, BoneXpert version 3 derives the BA from 21 bones, while version 2 used 13 bones, and version 3 also uses more advanced image analysis methods. To quantify this effect, we analyzed our dataset with the quartet method with version 2, yielding a precision of 0.12 years, i.e., an increase of 0.04 years. Thirdly, the old images from films had poorer image quality than the new images, which were recorded using digital radiography. The old films had been stored between 35 and 50 years before they were scanned in 2008.

The precision of cortical thickness T was found to be 23 microns. This is much smaller than the pixel size 170 microns of the 150 dpi images used by BoneXpert for the image analysis. This sub-pixel precision is obtained by forming T as the average of cortical thickness measured at many locations on the cortex, as illustrated in Fig. 2.

The treatment response in BA of the tubular bones after 2 years was 1.4 years, while in carpal BA it was only 1.0 year. This larger bone age advancement in phalanges compared to carpals in boys with KS treated with oxandrolone suggests a differential response to androgen stimulation in different bone types, potentially due to factors such as differential growth plate senescence, androgen receptor expression, local signaling pathways, and blood supply. This finding implies a need to consider the potential for premature growth plate closure and its impact on adult height when administering oxandrolone to boys with KS^18^. Since pediatric endocrinologists are most often looking for the effect of sex steroids, their focus is on BA of tubular bones, and this is the reason that BoneXpert reports BA separately for the tubular and carpal bones. Tubular bones are also known to be the relevant bones for adult height prediction, which is why Tanner replaced the TW2 BA based on tubular and carpal bones with the TW3 BA based only on tubular bones^19^.

The oxandrolone treatment increased height, suggesting a taller adult stature, but it also accelerated skeletal maturation, which has the opposite effect on adult stature. The net effect was that the predicted adult height increased by 2.5 cm in the treated group relative to placebo. A more precise BA measurement yields a correspondingly more precise adult height prediction, which is relevant for any condition in which predicted adult height is used to guide treatment decisions. However, care should be taken with this inference, because the BoneXpert adult height prediction model—as well as the older Bayley-Pinneau model^20^—was designed to model growth in untreated, non-pathological subjects, so this effect should be confirmed by looking at the *obtained* adult height, which was unfortunately not available in this study.

Hand X-rays of children with KS appear radiographically normal; radiologists do not typically attempt to diagnose KS from the hand X-ray alone. This is a critical observation for interpreting the generalizability of the present findings. The precision of BoneXpert depends on the quality of the automated bone border detection, which in turn depends on the bones having a normal radiographic appearance and on the hand being positioned flat on the detector. The majority of conditions encountered in pediatric endocrinology—including precocious puberty, GH deficiency, constitutional delay of growth, and Turner syndrome—similarly present with radiographically close to normal hand anatomy. The precision reported here is therefore expected to apply broadly across these conditions. Exceptions would include disorders that alter the radiographic appearance of the hand, in particular skeletal dysplasias, and conditions such as cerebral palsy in which the hand cannot be positioned flat on the detector; in such cases, poorer precision should be anticipated.

### Clinical implications of the found precision

1. BA: The smallest detectable change of BA was 2√2 *p* = 0.23 years. After 6 months of treatment, the treated participants in our sample had advanced 0.24 years on average relative to the placebo group, which implies that a repeat BA assessment after 6 months could detect this expected difference.
2. As established above, the precision of automated BA assessment is expected to generalize to most conditions in pediatric endocrinology, including central precocious puberty (CPP). In CPP, GnRH agonist treatment aims to halt BA progression, and the conventional follow-up interval is 12 months. The precision of 0.08 years reported here implies that automated BA can make such 12-month follow-up assessments considerably more informative: rather than merely detecting whether treatment has had an effect, the clinician can assess the degree to which BA progression has been suppressed. A shorter follow-up interval of 6 months would be technically feasible given the detectable change of 0.23 years, but published data suggest that the deceleration of BA progression is modest in the first 6 months of GnRH agonist treatment^21^, likely limiting the practical value of a 6-month assessment to research settings. In addition, the good precision is premised on using the same equipment for the follow-up (see Limitations below).
3. BHI: The precision of BHI was found to be 1.6%, and since the BHI reference curves used to derive BHI SDS entail a SD of 7.5%, constant across the bone age range, BHI SDS has a precision of 0.21 units, which multiplied by 2√2 yields 0.60 as the smallest detectable change in BHI SDS. Table 4 shows that the average treatment effect on BHI SDS after 1 and 2 years were 0.46 and 0.57 respectively, so it is not effective to use BHI SDS for monitoring oxandrolone treatment—the expected effect is too small to be detectable, which is also illustrated in Fig. 5.
4. The BHI SDS precision of 0.60 reported here is similarly applicable to other conditions in which BHI changes are of clinical interest. For example, in GH treatment of GH deficiency, first-year changes in BHI SDS of 0.80 have been reported^22,23^, exceeding the smallest detectable change of 0.60 and suggesting that automated BHI monitoring could detect such treatment effects within one year.
5. Limitations: The minimal detectable differences of 0.23 y for BA and 0.60 for BHI SDS are only validated when the *same* equipment is used to record the baseline and follow-up X-ray images as was the case in this study. It is also important that the software associated with the modality uses the *same* type of image postprocessing (edge enhancement). Requiring the use of the same equipment can be a challenge in hospitals with several X-ray rooms, as this may lead to inflexibility in the workflow. It is also important to standardize how the hand is posed on the X-ray detector, e.g. with a visual guide to the radiographers. It must be understood that the measurement chain is no stronger than the weakest link. Another limitation of the study is that it was done in children with a specific sex and pathology. Application in other patient groups might yield a poorer precision, if the children have more abnormal anatomy or if there are challenges placing the hand flat on the detector as in children with cerebral palsy.

The demonstrated precision may support value-based arguments for adoption of automated BA assessment in clinical practice.

## Conclusion

The use of serial BA measurement during treatment has rightfully been criticized to be imprecise due to the rater variability, estimated to be of the order 0.63 years^11^, rendering BA increment assessments of limited value. This study has shown that automated BA assessment drastically improves this situation. In addition to the accuracy error that was previously reported, we found a low precision error of 0.08 years implying that a BA change of 0.23 year is detectable. This has implications in both research and clinical contexts. In research, greater precision reduces the sample size needed to detect a significant difference or increases the power. In a clinical context, the improved precision reduces the time interval needed to appreciate a difference, allowing clinical decisions to be made sooner. This increases the clinical value in a follow-up after 1 year of treatment for children being managed for disorders of growth and puberty.

## Data Availability

The datasets generated during the current study are available from the last author (JR) on reasonable request.
